# Can flow cytometry be an alternative method for the detection of antimicrobial resistance?

**DOI:** 10.4314/ahs.v24i3.8

**Published:** 2024-09

**Authors:** Ozel Yuruker, Emrah Güler, Kaya Süer, Meryem Güvenir

**Affiliations:** 1 University of Kyrenia, Faculty of Medicine, Department of Immunology, Kyrenia, Cyprus; 2 European University of Lefke, Faculty of Arts and Sciences, Department of Molecular Biology and Genetics, Lefke, Cyprus; 3 Near East University, Faculty of Medicine, Department of Infectious Diseases and Clinical Microbiology, Nicosia, Cyprus; 4 Cyprus Health and Social Sciences, Faculty of Medicine, Department of Medical Microbiology, Guzelyurt, Cyprus

**Keywords:** Antibiotic resistance, ESBL, flow cytometry, MDR

## Abstract

**Background:**

The inappropriate use of antibiotics may cause antibiotic resistance, cause side effects, and eventually cause an increase in healthcare costs. This study aimed to determine a flow cytometry-based test to detect ESBL *E. coli* and MDR *P. aeruginosa* in a short time.

**Methods:**

The study included 25 *E.coli* isolates and 25 *P.aeruginosa* isolates identified by Phoenix TM 100 automated system [Becton Dickinson, USA]. In the flow cytometric method, the percentages of death cells exposed to cephalosporin including ceftazidime [CAZ] or cefotaxime [CTX] and clavulanic acid [CLA] combination, were compared with death cells exposed only to a cephalosporin [CAZ or CTX]. CLA index values [CAZ-CLA and CTX-CLA indices] were obtained for CTX and CAZ. Index values that were higher than 1.5 just for one cephalosporin were accepted as positive.

**Results:**

Cell viability was performed after 1-hour exposure to the drugs. When PI staining was applied to ESBL-treated and MDR-treated bacteria, 65% and 85% had nonviable membranes, indicating the method efficiently identified only cells with damaged membranes.

**Conclusion:**

Flow cytometry is a rapid and reliable method for the detection of ESBL and MDR in clinical microbiology laboratories

## Introduction

Rapidly, treating bacterial infections with appropriate antibiotic(s) is of major importance. The inappropriate use of antibiotics also causes antibiotic resistance, decreases their effectiveness 1, and may cause side effects which are one of the major issues because of the lack of discovery of new antibiotics. Consequently, inappropriate antibiotic treatments are significantly contributing to an increase in healthcare costs 2.

Faster and more accurate antibiotic susceptibility tests can significantly reduce morbidity and mortality rates as well as can be cost-effective. Due to the high mortality rate, especially in bloodstream infections caused by gram-negative bacteria, it is important to make an appropriate evaluation and plan.

The inappropriate use of empiric antibiotics was reported in a systematic review and meta-analysis ranging from 14% to 79%^3^. Although the patients receive an appropriate second-line antibiotic treatment afterward, empiric antibiotic treatments increase morbidity and mortality rates^3^-^5^. The use of carbapenems as an empirical treatment option in departments with critically ill patients has adverse effects on the hospital flora. Early detection of microorganisms that do not produce this enzyme and are sensitive to cephalosporins will prevent microorganisms such as carbapenemase-producing *Escherichia coli* (*E.coli*) from concentrating, colonizing, and causing nosocomial outbreaks in the hospital flora. Also, In recent years, globally, clones of multidrug-resistant (MDR) Pseudomonas aeruginosa (*P. Aeruginosa*) known as high-risk strains have become a health threat^6^. Additionally, the World Health Organization (WHO) indicated that carbapenem-resistant *P. aeruginosa* was reported into the “critical” group for which new antibiotics were urgently required 7. Therefore, rapid identification will ensure that the patient receives the appropriate treatment as soon as possible and will also prevent possible outbreaks in the hospital with the isolation of patients 8.

Flow cytometry; is a method that allows rapid optical analysis of cell populations on a single-cell basis. Flow cytometry has been used in microbiology since the mid-1970s 9. To overcome this difficulty, flow cytometry with cell viability markers could be used as a faster way over the classical method to detect changes in bacterial physiology caused by antibiotics. One of the advantages of this method is that some of the physiological changes caused by antibiotics can be seen before there is any growth inhibition.

The study aimed to determine a flow cytometry-based test which provides to detect ESBL-producing *E. coli* and MDR *P. aeruginosa* in a short time.

## Methods

### Bacterial Strains

The study included 25 E.coli isolates (20 ESBL positive and 5 ESBL negative E.coli) and 25 *P. aeruginosa* isolates (20 MDR positive and 5 MDR negative *P. aeruginosa*) identified by the Phoenix TM 100 automated system (Becton Dickinson, USA) in the Medical Microbiology Laboratory of Near East University Hospital between 2019-2020 were included. *E.coli* ATCC 25922 and *P. aeruginosa* ATCC 27853 standard strain was used as negative control.

### Preparation of Bacterial Strains for AST by Flow Cytometry

A working solution was prepared at a concentration of 0.1 mg/ml for cefotaxime (CTX) (Sigma, USA), 0.4 mg/ml for ceftazidime (CAZ) (Sigma USA), and 0.1 mg/ml for clavulanic acid (CLA) (Sigma, USA). Bacterial isolates stored at −80°C were recultured into the 5%'lik sheep blood agar at 35°C for 24 hours. And then, strains were inoculated into bottles containing 4 ml trypticsoy broth. The bottles were incubated at 35 °C for 60-75 minutes. Strains reaching 0.5 McFarland turbidity were prepared in the four tubes with 1/100 dilutions of bacterial suspension into 2 ml sterile trypticsoy buyyon. Added 80 µl CTX in the first tube, 80 µl CTX and 80 µl CLA in the second tube, 80 µl CAZ in the third tube, and 80 µl CAZ and 80 µl CLA in the fourth tube. All tubes were incubated at 35 °C for one hour.

### Staining Procedure For Flow Cytometry

For cultured bacteria, an approximate concentration range of 1×106 to 2×10^6^ bacteria/mL was diluted into a staining buffer. 5.0µl of Thiazole orange (TO) and 5.0µl of Propidium iodide(PI) solution were added to 500µl of cell suspension. The final staining concentrations were 420nmol/L for TO and 43µmol/L for PI. The solution was vortexed and incubated for 15 minutes in a dark place at room temperature.

### Flow Cytometry Analysis

All the calibration procedure was applied before the experiment as required.

The photomultiplier tube (PMT) voltages and the fluorescent compensations were adjusted as follows as directed by the manufacturer's instructions 10;

- Threshold-SSC and FL1
- FSC-E01, Logarithmic amplification
- SSC-375 V, Logarithmic amplification
- FL1-600 V, Logarithmic amplification
- FL3- 800 V, Logarithmic amplification
- Compensation- none used

After adjustments, the prepared samples were acquired on BD FACS Calibur using the SSC threshold. The obtained data were then analyzed using BD CellQuest Pro software in acquisition-to-analysis mode.

### Evaluation of Flow Cytometry Results

The percentage of dead cells that lost the wall integrity and took the dye into the cell and gave fluorescence in the total cells was determined. As a result of the examination, the percentages of cells that died when treated with the cephalosporin + CLA combination [CAZ+LA or CTX+CLA] were compared to the percentages of cells that died after treatment with only cephalosporin [CAZ or CTX]. CLA index value (CAZ-CLA and CTX-CLA) was obtained for CAZ and CTX. An index value greater than 1.5 for at least one cephalosporin was considered ESBL positive 11.

### Statistical analysis

Statistical analysis of the data obtained was conducted with SPSS (Statistical Package for the Social Sciences) Demo Ver 22.0 (SPSS Inc., Chicago, IL, USA) program. Person Chi-Square and Fisher's Exact test were used to determining statistical significance and the significance was evaluated at p <0.05.

## Results

**Figure 1:**
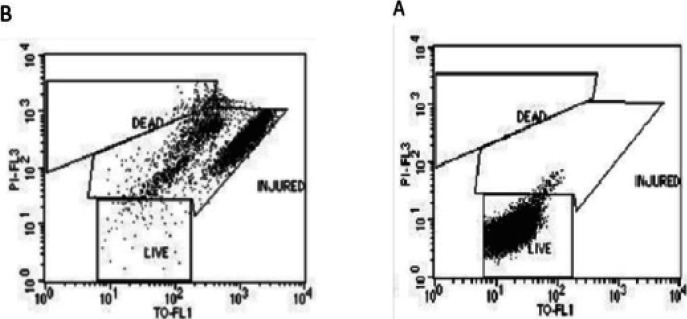
Flow Cytometry Images. A: Resistance Strain (Death/Injured;7.72%, Live;72.08%). B: Sensitive Strain (Death/Injured; 83.04%, 15.00%)

In our study, according to the phenotypic characteristics of ESBL-positive *E.coli* isolates, the mean CTX-CLA index was found to be 3.57, and the mean CAZ-CLA index was 2.19 (Figure 1). When the CTX-CLA and CAZ-CLA indexes for ESBL-positive E.coli isolates were compared by statistically, it was seen that the ability of the CTX-CLA and CAZ-CLA index to detect ESBL positives was the same. In addition, the ESBL positive detection rate was found to be 100% by using both indexes. The ESBL positive accuracy rate of the CTX-CAZ and CAZ-CLA index was found to be 65% [13/20] and 60% [12/20], respectively.

For MDR *P.aeruginosa* strains, the mean of the CTX-CLA index was found to be 2.47, and the mean of the CAZ-CLA index was 1.44. When the CTX-CLA and CAZ-CLA indexes for MDR *P.aeruginosa* isolates were compared by statistically, it was seen that the ability of the CTX-CLA and CAZ-CLA index to detect MDR-positive *P. aeruginosa* was the same. The MDR positive accuracy rate of the CTX-CAZ and CAZ-CLA index was found to be 85% [17/20] and 75% [15/20], respectively.

## Discussion

One of the major challenges is to treat bacterial infections promptly with the appropriate antibiotic(s). The use of inappropriate antibiotic treatments causes higher morbidity and mortality rates, even if the patient receives appropriate antibiotic treatment as a second line of treatment. The inappropriate use of antibiotics also increases the risk of antibiotic resistance, decreases their effectiveness, and causes side effects, causing a significant increase in healthcare costs.

In hospitals, antibiotics are the most commonly used group of drugs. Standard methods of testing antibiotic sensitivity are labor-intensive and time-consuming, taking up to 24 hours, the analysis typically lasts up to 72 hours 12.

The failure in rapid and reliable detection of ESBL-producing isolates may also compromise infection control measures, which further contributes to its spread. Since confirmation methods are often necessary, detecting ESBLs rapidly may not be easy and readily possible during routine susceptibility testing. Clinical laboratories use a variety of semi-automated systems to detect ESBLs. Traditionally, this detection has always been based on growth assessment in the presence of cephalosporins with and without CLA.

Flow cytometry has begun to be used in bacteriology for both the rapid identification of bacteria and the determination of antibiotic susceptibility.

Flow cytometry has the advantage of being able to measure these parameters quickly and provide quantitative results.

Therefore, it may be reliable and faster to perform the total bacterial count using flow cytometry. The antibiotic sensitivity of bacteria has been investigated extensively by analyzing the viability of the bacteria, its DNA content, its effect on the cell membrane, antibody binding, metabolic effects, and protein content 13. Here, the antimicrobial susceptibility of bacterial cells was assessed using flow cytometry, and we used PI/TO staining over other stains because it can be easily found in most laboratories and is inexpensive. By exposing clinical isolates of ESBL and MDR [resistant or susceptible] to different antimicrobial drug classes and then staining them with the PI/TO viability marker, their fluorescence intensity could be measured. Thus, bacteria cells exposed to antimicrobial drugs were considered viable or nonviable according to their ability to allow the entrance of PI through the cell membrane.

Faria-Ramos et al. evaluated the flow cytometry method in 61 samples, 41 of which were ESBL positive and 20 ESBL negative, reporting that the method provides fast and accurate results^11^. Canturk et al. found that 38 ES-BL-positive and 10 ESBL-negative Enterobacteriaceae isolates identified by GST, disk diffusion, and automatic methods were used; it was found that the CTX-CLA and CAZ-CLA indexes were significantly higher in ES-BL-positive isolates compared to ESBL-negative isolates 13. When both indexes were compared, it ws found that the identification characteristic of the CTX-CLA index was higher^14^. In another study, The cytometry method showed acceptable results on the E. coli model. The relative accuracy was 88.8%, the sensitivity was 85.7%, the specificity was 88.8%, and the agreement test showed 0.524, which is an average value of 12. In our study, the ESBL positive detection rate was found to be 100% by using both CTX-CAZ and CAZ-CLA.

As a Gram-negative pathogen, *Pseudomonas aeruginosa* also poses a significant risk for infection and contributes to significant morbidity and mortality in humans. As one of the most common and severe causes of hospital-acquired infections, it especially affects immunocompromised patients and intensive care unit [ICU] patients The majority of *P. aeruginosa* strains are resistant to most antibiotics currently available 15. *P. aeruginosa*, commonly known as MDR/XDR, has become a public health threat due to the widespread of ‘high-risk clones’ that contain multiple or extensive drug resistance. Epidemiology and treatment of multidrug-resistant and extensively drug-resistant *Pseudomonas aeruginosa* infections^6^. Our objective was also to analyze MDR and our findings indicated that using flow cytometry, the positive accuracy rate of CTX-CAZ and CAZ-CLA indexes were 85% [17/20] and 75% [15/20], respectively, for MDR *P.aeruginosa*.

Compared with the incubation period of GST, disk diffusion, and automatic methods for at least one night, the current cytometry method can quickly and reliably identify ESBL in as little as two hours. As a result, it is considered that the current cytometry method can be a fast and reliable method for the detection of ESBL in clinical microbiology laboratories 14. Both pseudo-positive and pseudo-negative results have been observed. The method under discussion has generally shown good agreement with the commonly accepted Gold standard method, the delusion test. It was found that there was no significant difference in accuracy, specificity, or sensitivity depending on the culture examined. In terms of negative aspects, even though the tube test is less labor-intensive and less time-consuming than the delusion test, it is not as advantageous as the disc-diffusing method. Nonetheless, the method's reduction of time (2.5 vs. pure culture isolation) is an advantage 12.

We tested two classes of antibiotics routinely used in hospitals for their antimicrobial susceptibility. Cell viability was performed after 1-hour exposure to the drugs. When PI staining was applied to ESBL-treated bacteria and MDR-treated bacteria, 65%, and 85%, respectively, had nonviable membranes, indicating the method was efficient in identifying only cells with damaged membranes.

The limitation of this study is that flow cytometry would not replace classical bacterial susceptibility testing since most laboratories lack adequate equipment and the use of flow cytometry may not be appropriate for evaluating antibiotics that damage bacteria DNA, which has become refractory to PI.
